# Editorial: Pathogenic mechanism and biocontrol of *Xanthomonas* on plants

**DOI:** 10.3389/fcimb.2023.1270750

**Published:** 2023-08-18

**Authors:** Yangyang Zhao, Pedro Laborda, Sang-Wook Han, Fengquan Liu

**Affiliations:** ^1^Institute of Plant Protection, Jiangsu Academy of Agricultural Sciences, Nanjing, China; ^2^School of Plant Protection, Key Laboratory of Green Prevention and Control of Tropical Plant Diseases and Pests, Ministry of Education, Hainan University, Haikou, China; ^3^School of Life Sciences, Nantong University, Nantong, China; ^4^Department of Plant Science and Technology, Chung-Ang University, Anseong, Republic of Korea

**Keywords:** *Xanthomonas*, diversity, type IV secretion system (T4SS), quorum sensing (QS), biocontrol, Bacillus, Essential oils (EOs)

*Xanthomonas*, a Gram-negative bacterium, belonging to the class Gammaproteobacteria, can infect more than 400 different plants including a wide variety of important crops such as rice, wheat, citrus, tomato, pepper, cabbage, banana, and bean (An et al., 2020 and Timilsina et al., 2020). Some devastating diseases caused by *Xanthomonas* have been reported from multiple important crops worldwide. In the top 10 plant pathogenic bacteria, the fourth, fifth, and sixth positions are *Xanthomonas* species (Mansfield et al., 2012). For example, *X. oryzae pv. oryzae* (*Xoo*) and *X. oryzae pv. oryzicola* (*Xoc*) are the causal agents of bacterial blight and bacterial leaf streak of rice, respectively, which are two severe diseases affecting rice production and quality in tropical and subtropical regions. Moreover, *X. campestris* pathovars cause diseases in a range of crops. The articles in the Research Topic on the pathogenic mechanisms and biocontrol of *Xanthomonas* on plants explore the diversity, virulence factors, and potential management of *Xanthomonas*. The results provide a broader insight into *Xanthomonas* pathogenicity in relation to host specificity and spread, and the alternative protection strategies of plants.

*Xanthomonas* diversity has been largely investigated through genome sequencing and characterized using advances in omics tools (An et al., 2020 and Timilsina et al., 2020). *Xoo* causes bacterial blight disease of rice (*Oryza sativa*), which is one of the major diseases affecting rice production. Song et al. employ whole-genome sequencing to explore the diversity and evolution of *Xoo* in the main rice-growing areas of China over the past 30 years. They reveal six lineages including CX-1 to CX-6, in which CX-5 and CX-6 were the most prevalent across all studied areas. Recent sporadic disease outbreaks have been primarily caused by *Xoo* isolates derived from lineages CX-5 and CX-6. The rapid virulence evolution of *Xoo* against rice is analyzed using large-scale virulence tests and is correlated to the genetic background of *Xoo*, rice resistance genes, and the planting environment of rice. The population genomic study together with a large-scale virulence evaluation of *Xoo* may help build durable resistance and management strategies against this pathogen.

Bacterial type IV secretion (T4S) systems are multiprotein complexes that deliver DNA, effectors, and protein–DNA complex to the extracellular milieu or into the eukaryotic and prokaryotic target cells (Costa et al., 2021). Drehkopf et al. demonstrate the existence of the T4S systems in the tomato and pepper pathogen *X. euvesicatoria*, which is the only known plant pathogen with a VirB/VirD4- and an Icm/Dot-like T4S system. The VirB/VirD4 T4S system acts as a conjugation system for plasmid transfer between *X. euvesicatoria* strains and shares substrate specificity with the Icm/Dot system which serves as an additional protein delivery system. VirB/VirD4 T4SS in *X. citri* provides strains with the ability to kill other Gram-negative bacteria in a contact-dependent manner (Souza et al., 2015), but there is no toxic effect of T4S systems in *X. euvesicatoria* on other bacteria. The study provides a perspective on the function of T4S systems in *X. euvesicatoria*, which helps us understand more about this plant pathogen.

The biocontrol strategies used for *Xanthomonas* increasingly depend on the application of microbial biocontrol agents, or microbiome engineering. The diffusible signal factor (DSF) family is an important type of quorum sensing (QS) signal found in diverse Gram-negative bacteria and mediates intraspecies, interspecies, and inter-kingdom communication (He et al., 2023). It has been reported that DSF in *X. campestris* elicits innate immunity in plants and is suppressed by the exopolysaccharide xanthan (Kakkar et al., 2015). However, another study showed that DSF produced by *X. campestris* pv. *campestris* (*Xcc*) can suppress pathogen-associated molecular pattern-triggered immunity (PTI) in *Arabidopsis thaliana* (Tran et al., 2020). Zhao et al. report that a low concentration (1–5 μM) of DSF could prime plant immunity against *Xcc* when interacting with plants and is mediated by the jasmonic acid signaling pathway. The finding provides new insight into the function of DSF and an alternative strategy for the control of black rot in plants.

*Bacillus* strains are always effective and reliable alternatives to develop as a microbial pesticide to fight against *Xanthomonas* (Marin et al., 2019). Zhou et al. report that *Bacillus velezensis* strain 504 exhibits apparent antagonistic activity against *Xoc* wild-type strain RS105, and is a potential biocontrol agent for bacterial leaf streak, exhibiting relative control efficiencies over 70% on two susceptible cultivars. Approximately 77% of the *Xoc* RS105 genes are differentially expressed in the presence of cell-free supernatants of *B. velezensis* 504, which sheds light on the mechanisms of biological control agent-impaired *Xoc*.

Essential oils (EOs) or EO-based products are promising candidates for being used as biocontrol agents due to their broad-spectrum activity against fungi, bacteria, viruses, pests, and weeds, and are environmentally friendly and economically viable (Chang et al., 2022). EOs have been isolated from citrus cultivars to inhibit the *X. citri* subsp. *citri* that causes citrus bacterial canker (Mirzaei-Najafgholi et al., 2017). Nagy et al. show that EOs, especially cinnamon, are effective against *X. arboricola* pv. *pruni* (*Xap*), which causes bacterial spots on stone fruits. They also report that direct bioautography is a fast and suitable method for screening anti-*Xap* components of complex matrices. The study offers a possibility that EOs could be used to control plant disease caused by *Xap*.

*Xanthomonas* spp. cover a variety of plant pathogens that utilize a wide range of virulence factors for pathogenicity and fitness in plant hosts. This Research Topic presents the latest research and perspectives on the diversity and pathogenicity of *Xanthomonas* and its potential biological control agents. This Research Topic will provide researchers with the opportunity to advance perspectives on the pathogenic mechanism and biocontrol of *Xanthomonas*.

## Author contributions

YZ: Writing – original draft. PL: Writing – review & editing. S-WH: Writing – review & editing. FL: Funding acquisition, Supervision, Writing – review & editing.
